# Structural and Molecular Features of Human Extraocular Muscle Spindles Suggest a Unique Role in Proprioception

**DOI:** 10.1167/iovs.67.11.27

**Published:** 2026-09-15

**Authors:** Arzu Petersen, Paata Pruidze, Giorgi Didava, Atieh Seyedian Moghaddam, Stefan H. Geyer, Génova Carrero-Rojas, Angel M. Pastor, Wolfgang J. Weninger, Roland Blumer

**Affiliations:** 1 Division of Anatomy, Center for Anatomy and Cell Biology, Medical University of Vienna, Vienna, Austria; 2 Departamento de Fisiología, Facultad de Biología, Universidad de Sevilla, Sevilla, Spain

**Keywords:** proprioception, extraocular muscles (EOMs), human extraocular muscle spindles, vesicular glutamate transporter, exocytotic protein

## Abstract

**Purpose:**

Muscle spindles in extraocular muscles (EOMs) of humans exhibit atypical features, putting their function into question. We tested the presence of molecules, previously described in skeletal muscle spindles, within the human EOM spindles. We also investigated the anatomic trajectory of proprioceptive fibers within the EOM motor nerves.

**Methods:**

The EOMs and the EOM nerves were obtained from three individuals (mean age of 84.7 ± 13.6). We also used data from two separate studies, including the EOMs of a 2-year-old child and pig EOMs. For the analyses, we integrated multi-color immunofluorescence, confocal 3D reconstructions, and transmission electron microscopy (TEM).

**Results:**

Human EOM spindles contain nuclear chain fibers and intrafusal fibers that resemble extrafusal fibers. Only nuclear chain fibers express myosin light chain 2. The spindle capsule expresses glucose transporter 1. Sensory nerve terminals of the muscle spindles contain clear vesicles, which is confirmed through synaptophysin expression. These terminals also express vesicular glutamate transporter, indicating storage of the neurotransmitter glutamate. Furthermore, sensory nerve terminals express syntaxin, complexin, and synaptobrevin, proteins involved in neurotransmitter release. Sensory (proprioceptive) fibers are not present at the exit site of the EOM motor nerve from the brainstem. They merge with the EOM motor nerves at the superior orbital fissure.

**Conclusions:**

Our study has uncovered glutamatergic machinery in human EOM spindles, analogous to that of skeletal muscle spindles. These findings do not support the hypothesis that human EOM spindles are vestigial. Instead, they suggest that spindles are functional, providing feedback about the eye's position.

Gaze direction provides the essential context needed to interpret visual information. It is supposed that the brain calculates the eye position by integrating corollary discharge signals with proprioceptive feedback from various sources,^1^ including the extraocular muscles (EOMs).^2^^,^^3^ Several studies provide evidence that the brain receives eye position signals,^4^^–^^7^ and Goldberg's laboratory has found a tonic eye position signal in area 3 of the somatosensory cortex of monkeys.^8^ However, the source of proprioceptive feedback remains unclear. This lack of knowledge is disadvantageous, as EOM proprioception is crucial for the development of binocular vision.^9^^,^^10^

The principal proprioceptor in skeletal muscles is the muscle spindle, which is an encapsulated structure that contains nuclear chain and nuclear bag fibers, and which receives both sensory and motor innervation.^11^^–^^14^ Muscle spindles are also present in human EOMs.^15^^–^^17^ However, their limited periaxial space, the absence of nuclear bag fibers, and the presence of both nuclear chain fibers and fibers with characteristics of extrafusal fibers within the spindle capsule are noteworthy features.^15^^–^^18^ Intrafusal fibers resembling extrafusal fibers were designated as anomalous fibers.^17^ These structural particularities have been identified in EOM spindles of aged persons^16^^,^^17^ and infants,^15^ and so are not attributable to age-related changes. The innervation of human EOM spindles also exhibits particularities. Specifically, not all intrafusal fibers receive sensory innervation,^15^^,^^17^ and although motor terminals are present in a 2-year old child,^15^ they have not been documented in aged persons.^17^ The atypical morphology of human EOM spindles has led to an ongoing debate whether they are a source of proprioceptive input or just vestigial structures.^2^^,^^3^^,^^15^^,^^16^ Clearly, it is imperative to enhance our understanding of this structure.

In recent years, distinct tissue molecules and neuronal molecules have been identified in skeletal muscle spindles, thereby helping to define their structure and function.^11^^,^^19^^,^^20^ Such molecular information is missing in human EOM spindles, indicating that key features of these organs are still unknown. Our work here determined a molecular profile of human EOM spindles comparable with the canonical skeletal spindles.^11^^,^^19^^,^^20^ We also determined that proprioceptive fibers do not travel into the brain using the EOM motor nerves.

## Materials and Methods

Tissue was collected from three human body donors (3 female cadavers), enrolled in the body donor program of the Medical University of Vienna in Austria. They had provided voluntary, written consent for their bodies to be used for teaching and research. In addition, the study was approved by the Ethics Committee of the Medical University of Vienna (Ethics Commission Number 1183/2025). All methods adhered to the general rules and regulations of the Medical University of Vienna and complied with the tenets of the Declaration of Helsinki. The age of the body donors was 69, 92, and 93 (mean value = 84.7 ± 13.6). Only body donors arriving at the Division of Anatomy within 6 to 8 hours after their death were included.

In addition, further data used in this study came from previous studies on EOMs of a 2-year-old child^15^ and a pig,^21^ which both received ethical approval. Please see respective publications for further information.

### Tissue Collection

The skulls of fresh body donors were opened and the brains were extracted. Then, the roof of the orbit was removed, and the eyeballs, along with the associated eye muscles, were extracted from the orbital cavity. In addition, 1-cm long segments of the oculomotor (CN III), trochlear (CN IV), and abducens nerves (CN VI) were biopsied each at three positions: position 1 was immediately after their exit from the brain stem, position 2 was at the mid-level of the cavernous sinus, and position 3 was at the superior orbital fissure.

Following dissection, the eyeballs with muscles and the nerve samples were fixed in 4% paraformaldehyde in 0.1 M phosphate buffer saline (PBS) at a pH 7.4 for 24 hours at 4°C. For all collected tissues the immersion fixation started within 7 to 10 hours postmortem. This brief postmortem interval ensures reliable immunohistochemical staining results from fresh human tissue.^22^ After fixation, the samples were placed in 0.1 M PBS pH 7.4 for 24 hours. The eye muscles were removed from the eyeballs, and all muscle and nerve samples were processed for cryo-sectioning.

### Tissue Preparation

For samples to be cryo-sectioned, tissue was cryoprotected using a stepwise protocol of increasing concentrations of sucrose (10%, 25%, and 40% dissolved in PBS) over 2 days. The sugar molecules provide multiple crystallization points, preventing large destructive crystals from forming and destroying cells. Prior to embedding, whole eye muscles were divided into three pieces (proximal, middle, and distal). Samples were embedded in a transparent cryomatrix embedding solution (Thermo Fisher Scientific, Waltham, MA, USA), frozen and stored at −80°C degrees. A Leica cryostat microtome, set at −20°C, was used for sectioning. Cross sections through the muscles were cut and examined under the light microscope. When muscle spindles were identified, sections at 10-µm thickness were cut until the end of the muscle spindle was reached. Sections were mounted on Thermo Fisher Superfrost Ultra Plus slides. Alternatively, horizontal sections 150-µm thick were cut parallel to the surface of the EOMs to obtain muscle spindle whole mount preparations. Whole mounts were transferred to well plates and further processed as floating sections. Cross sections and floating sections were used for multicolor immunofluorescence.

### Antibodies and Labeling Toxins

The following neuronal markers, tissue markers, and toxins were used. Neuronal markers included anti-neurofilament (NF) as a pan-neuronal marker, anti-choline acetyltransferase (ChAT) as a marker for motor neurons,^23^ and anti-tyrosine hydroxylase (TH) as a marker for postganglionic sympathetic axons.^24^ We used anti-synaptophysin (Syn)^25^ as a marker for nerve terminals and anti-synaptobrevin (SYB),^26^ anti-syntaxin (STX),^27^ and anti-complexin (CPLX)^28^ as markers for proteins involved in neurotransmitter release. Anti-vesicular glutamate transporter 1 (VGLUT1) was used as a marker for glutamatergic synapses.^20^ We used anti-glucose transporter 1 (GLUT1) as a marker for the perineurial capsule and anti-myosin light chain 2 (MYL2) as a marker for muscle fibers.^19^ Two anti-GLUT1 antibodies, from a rabbit and a mouse, were used, depending on the specific staining combination. Toxins included phalloidin (Phall) as a general marker for muscle fibers^29^ and ɑ-bungarotoxin (BTX) as a marker for motor nerve synapses.^30^ All primary antibodies and labeling toxins as well as their working dilution, RRID numbers, and suppliers are listed in the Table. Secondary antibodies were raised in a goat, rabbit, or donkey and conjugated with either Alexa Fluor (AF) 488, AF 568, or AF 647. These were obtained from Thermo Fisher Scientific (Waltham, MA, USA) and used at a concentration of 1:500.

**Table.** Markers Utilized in the Present Study, Including Working Dilutions, Catalog Numbers, and Suppliers

Markers	Working Dilution	Catalog/RRID Number	Supplier
Primary antibodies			
Neuromolecular targets			
Anti-neurofilament (NF)	1:2000	AB5539/AB_177520	Merck/Millipore
Anti-choline acetyltransferase (ChAT)	1:100	AB144P/AB_2079751	Merck/Millipore
Anti-tyrosine hydroxylase (TH)	1:400	AB152/AB_390204	Sigma Aldrich
Anti-synaptophysin (Syn)	1:300	MAB329/AB_95786	Merck/Millipore
Anti-synaptobrevin (SYB)	1:500	AB104002/AB_887807	Synaptic System
Anti-complexin (CPLX)	1:500	122003/AB_2619793	Synaptic System
Anti-syntaxin (STX)	1:500	110403/AB_887900	Synaptic System
Anti-vesicular glutamate transporter 1 (VGLUT1)	1:500	135303/AB_887875	Synaptic System
Tissue molecular targets			
Anti-myosin light chain 2 (MYL2)	1:200	AB79935/AB_1952220	ABCAM
Anti-glucose transporter 1 (GLUT1)	1:500	66290-1-Ig/AB2881673	Proteintech
Anti-glucose transporter 1 (GLUT1)	1:500	AB115730/AB115730	ABCAM
Toxins			
Phalloidin (Phall)	1:200	65906-10NMOL	Sigma-Aldrich
α-bungarotoxin (BTX)	1:500	B35450	Thermo Fisher

### Immunofluorescence Labeling

Protocols for immunolabeling of both 10 µm cryo-sections on slides and 150 µm floating sections are provided.

#### Immunofluorescence Protocol for Cryo-Sections

Depending on the staining combination, 10% blocking serum, from a rabbit, goat, or donkey was applied to the slides for 1 hour. Next, primary antibodies were diluted in PBS+0.1%-Triton X-100 (PBST) and applied to the slides for 24 hours at 4°C (except anti-ChAT, which incubated for 48 hours). After incubation, the slides were washed in PBS and stained with the appropriate secondary antibodies diluted in PBST for 2 hours at 37°C. Slides were then washed, counterstained with DAPI for 10 minutes, washed again, and mounted with fluorescent mounting medium (Dako). A cover slip was applied. All slides were stored at 4°C.

#### Immunofluorescence Protocol for Floating Sections

Floating sections were placed in multiwell plates and maintained in PBS+1%-Triton X-100 for 24 hours. Subsequently, the appropriate blocking serum was applied for 2 hours, followed by the primary antibody for 48 hours. Samples were washed in PBS for 5 × 10 minutes and subjected to secondary antibody solution for 4 hours. Then they were again washed, mounted with a PBS:Glycerol (40:60) mounting solution, and cover slipped.

### Imaging

After fluorescent labeling, the slides were immediately analyzed and imaged using a Confocal Laser Scanning Microscope (CLSM; Olympus FV3000; Olympus Europa SE & Co. KG, Hamburg, Germany). Three lasers with excitation wavelengths of 488 nm, 568 nm, and 647 nm were used to capture fluorescent-colored images. Brightfield images (using 647 nm excitation wavelengths) were also sometimes acquired to visualize structural features of the tissue. Depending on the region of interest, image sequences at 10×, 20×, 40×, and 60× magnifications were captured to create virtual z-stacks.

### Transmission Electron Microscopy

For electron microscopy, we used the EOMs from a 2-year-old multiorgan donor that we had used in a previous study.^15^ The study was performed in accordance with the Austrian federal transplantation law.

The EOMs were immersion-fixed in 2% paraformaldehyde and 2.5% glutaraldehyde in 0.1 M cacodylate buffer at pH 7.4 for 1 day. They were postfixed in buffered 1% osmium tetroxide, dehydrated in graded solutions of ethanol, and embedded in Epon. When muscle spindles were identified from semithin sections under the light microscope, ultrathin sections were cut, mounted on dioxane formvar-coated copper grids, contrasted in a solution of 2% uranyl acetate followed by a solution of 0.4% lead citrate, and examined under a transmission electron microscope (TEM, model EM9; Zeiss, Oberkochen, Germany). For a more detailed description see Blumer et al. 1999.^15^

### Three-Dimensional Image Reconstruction

For 3D reconstruction, confocal image stacks of human and pig EOM spindles, that had been labeled with anti-neurofilament and anti-synaptophysin antibodies were imported into Amira 2025.1 (Thermo Fisher Scientific Inc., Mérignac, France). Confocal stacks of the pig EOM spindles came from a separate study.^21^ Three-dimensional computer representations were generated using the volume rendering module.

### Data Analysis

Data storage, statistical analyses, and image analyses were performed in either SPSS (IBM SPSS Statistics version 29.0; IBM Corporation, Armonk, NY, USA) or Image J software.^31^ Statistical differences were calculated using either the independent samples *t*-test (for parametric data) or the Mann-Whitney *U* Test (for non-parametric data). Statistical significance level was set at *P* < 0.05.

## Results

A total of 54 muscle spindles were analyzed, including 25 spindles from vertical EOMs (inferior rectus muscle), 24 spindles from horizontal EOMs (medial rectus), and 5 spindles from oblique EOMs (inferior oblique muscle). See Supplementary Table S1.

### Structural Features of Human EOM Spindles

**Figure 1.**
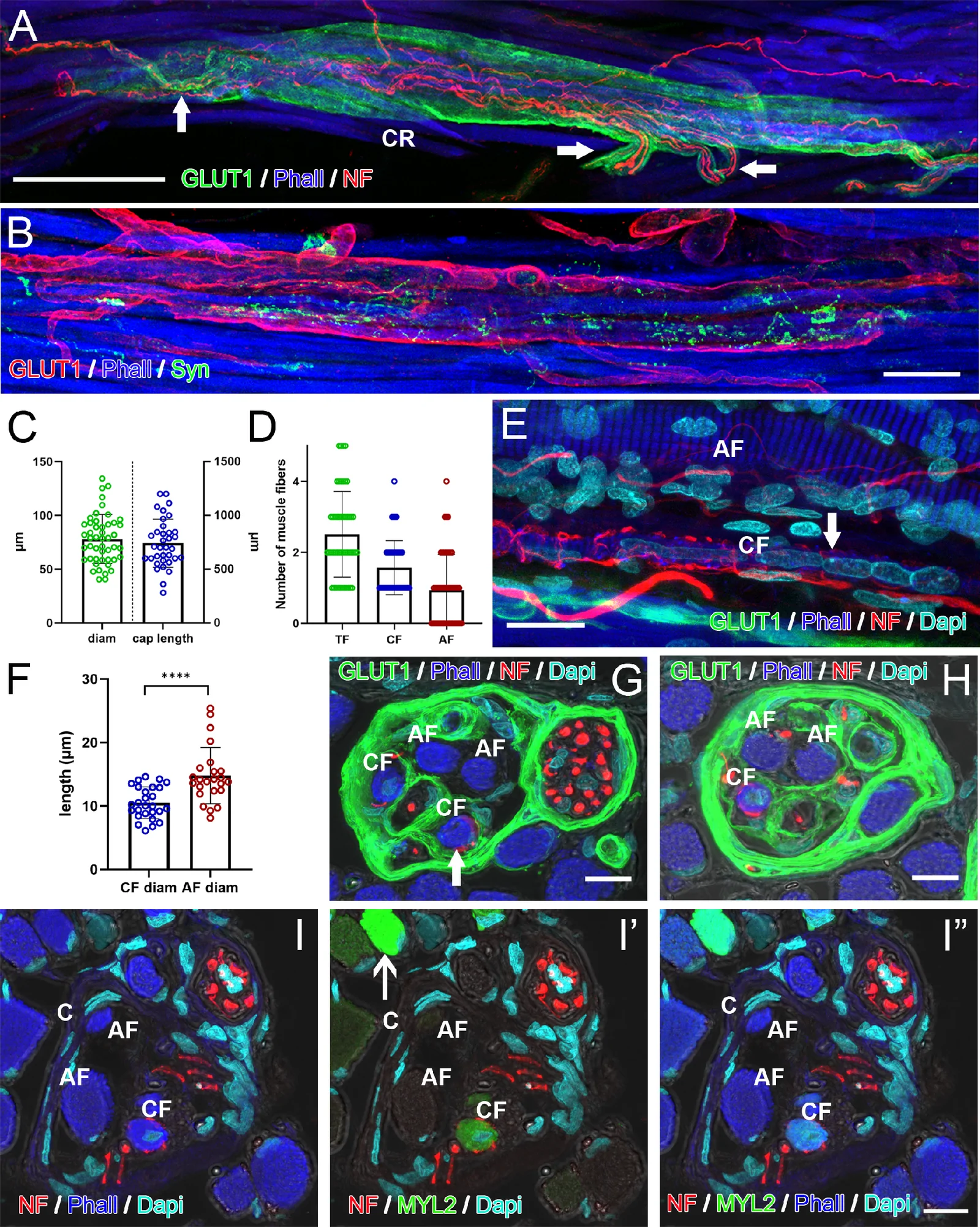
Morphological characteristics and tissue proteins of human EOM spindles. (**A, B**) Confocal microscopy images of muscle spindle whole mount preparations show that muscle spindles vary in shape. The spindle capsule (*green* in **A**, *red* in **B**) exhibits slight expansion **A** or no expansion **B** in the central region (CR). The arrows in **A** indicate nerve entry sites. In **A**, the spindle capsule (*green*), muscle fibers (*blue*), and nerve fibers (*red*) were labeled with anti-GLUT1, phalloidin (Phall), and anti-NF, respectively. In **B**, the spindle capsule (*red*) and muscle fibers (*blue*) are labeled with the same markers as in **A**, whereas anti-synaptophysin (Syn) displays nerve terminals (*green*). (**C, D**) Bar chart illustrating the mean maximum diameter and capsule length of the total number of muscle spindles **C**, and the mean value of total muscles fibers (TFs), as well as the mean value of nuclear chain fibers (CFs) and anomalous fibers (AFs) per spindle **D**. (**E**) Confocal microscopy image of a muscle spindle whole mount preparation, showing a CF with a row of central nuclei (*arrow*), and an AF with peripheral nuclei. Labeling with anti-GLUT1 (*green*), Phall (*blue*), and anti-NF (*red*). In these and other sections, the cell nuclei are labeled with Dapi (*light blue*). (**F**) Bar chart showing the mean maximum diameter of CFs and AFs. (**G, H**) Confocal microscopy images showing a fiber stopping in a muscle spindle. Cross sections in the central **G** and polar region **H** are shown, using the same staining combination as in **A***.* In the central region **G**, two CFs and two AFs are present, whereas in the polar region **H** one CF is absent, indicating that this fiber does not reach the end of the spindle. The arrow in **G** indicates the CF that does not reach the end. (**I–I”**) Confocal microscopy images of a cross-sectioned muscle spindle showing MYL2 immunoreactivity. A CF expressing MYL2 and AFs without MYL2 expression are shown. One extrafusal fiber (*thin arrow*) also expresses MYL2, exhibiting higher staining intensity than the CF. C, spindle capsule. Labeling with anti-NF (*red*), anti-MYL2 (*green*), and Phall (*blue*). Images are shown in split channels, with anti-NF along with phalloidin **I** or anti-MYL2 **I’**, and in the overlay **I”**. *Scale bars* = 200 µm in **A**; 100 µm in **B**; 25 µm in **E**; 15 µm in **G**, **H**, **I** to **I”**. Error bars in bar charts represent standard deviation.

Most muscle spindles were found within a transition zone between the orbital and the global layer. Other muscle spindles were within the global layer, close to the transition zone. These findings are consistent with those of a previous study on human EOM spindles.^16^ Most spindles had a fusiform shape, with variable expansions in the central region (Fig. 1A). Other spindles had a cylindrical shape, without expansions (Fig. 1B). The mean diameter of EOM spindles, measured in the central region, was 78 ± 22.8 µm, and the mean capsule length was 741.5 ± 224.5 µm (see the bar chart in Fig. 1C).

Muscle spindles contained between one and five intrafusal muscle fibers. A total of 44% had only one or two muscle fibers (see Supplementary Table S1). The mean number of intrafusal fibers was 2.5 ± 1.2 (see the bar chart in Fig. 1D). Two types of intrafusal muscle fibers were distinguishable: nuclear chain fibers and anomalous fibers, as termed by Ruskell.^17^ Nuclear chain fibers contained a chain of nuclei running through the center of the muscle fiber and had a mean diameter of 10.5 ± 2.5 µm. Anomalous fibers had peripheral nuclei and a significantly larger diameter (mean value = 14.8 ± 4.4, *P* value < 0.0001; Fig. 1E, bar chart in Fig. 1F). Every spindle contained at least one nuclear chain fiber (mean value = 1.6 ± 0.8), and zero to four anomalous fibers (mean value = 1.0 ± 1.0; see the bar chart in Fig. 1D). As previously reported,^15^^–^^17^ nuclear bag fibers, which are a feature of skeletal muscle spindles, were not present in our sample of human EOM spindles. In 18.3% of the spindles, there was abrupt cessation of intrafusal fibers before reaching the end of the spindle. This fiber stop occurred in both nuclear chain and anomalous fibers (Figs. 1G, 1H; Supplementary Fig. S1, which shows a comprehensive array of cross-sections through the same muscle spindle). The morphological features of all muscle spindles that were analyzed are displayed in the Supplementary Table S1.

### Human EOM Spindles Express the Tissue Proteins GLUT1 and MYL2

Immunolabeling with an antibody against GLUT1 showed that the outer capsule of the human EOM spindles expressed GLUT1 (see Figs. 1A, 1B, 1G, 1H). GLUT1 immunoreactivity was also present in fibroblasts, which surround intrafusal muscle fibers, and which form the inner capsule of the spindle (see Figs. 1G, 1H).

Labeling with an antibody against MYL2 showed that within the spindles only the nuclear chain fibers expressed MYL2, not the anomalous fibers. Furthermore, approximately ⅓ of the extrafusal muscle fibers also expressed MYL2. The signal intensity of MYL2 was higher in extrafusal fibers compared with nuclear chain fibers (Figs. 1I–I”).

### Sensory and Motor Components of Human EOM Spindles

To differentiate between motor and sensory nerve fibers, we performed double labeling with antibodies against NF and ChAT. ChAT is the synthesizing enzyme for the neurotransmitter acetylcholine, and it is expressed in motor, but not sensory neurons.^23^

**Figure 2.**
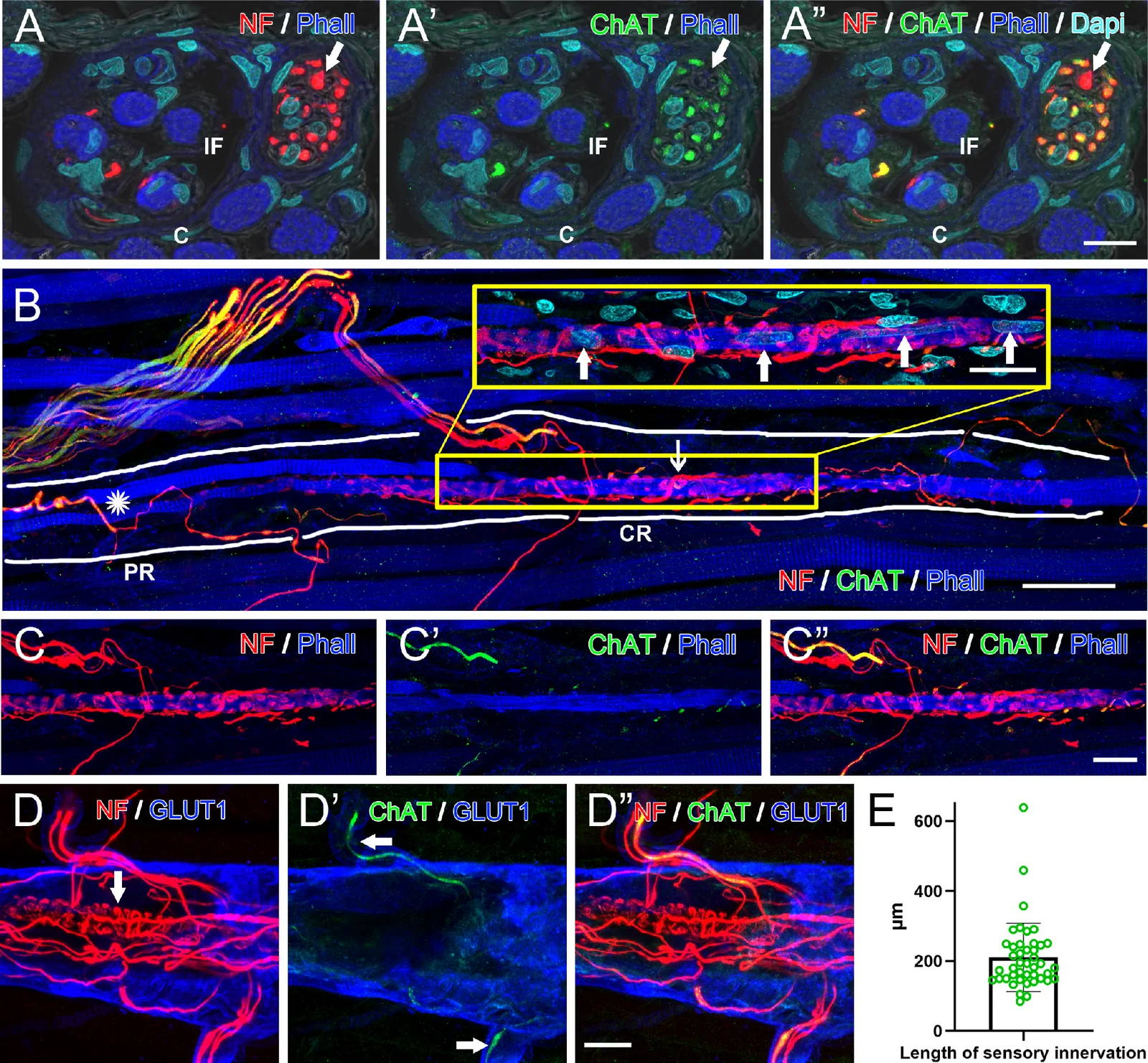
Sensory and motor innervation of the human EOM spindles. (**A–A”, B**) Confocal microscopy images show muscle spindles in cross sections **A** to **A”** as well as in whole mount preparation **B**. Labeling was with anti-NF (*red*), anti-ChAT (*green*), and phalloidin (*blue*). In **A**, channels are split and images are presented in NF/phalloidin **A** or ChAT/phalloidin labeling **A’** and in the overlay **A”**. **A** to **A”** Shows the nerve entry site, where almost all fibers are motor, as demonstrated by ChAT expression. Only a single large diameter fiber (*arrows* in **A–A”**) without ChAT signal is present, suggesting a sensory fiber nature. Spindle capsule (C), and intrafusal fibers (IFs). **B** Shows the entire muscle spindle. The *white lines* in **B** denote the spindle capsule, which remained unlabeled. Upon entering, a ChAT-negative sensory fiber (*small arrow* in **B**) coils around a nuclear chain fiber in the central region (CR), whereas a ChAT-positive motor fiber (*asterisk* on left side in **B**) extends toward the polar region (PR). The magnified inset in **B** shows the central nuclei (*thick arrows*) of the chain fiber. (**C – C”**) High magnification images of the boxed inset of **B***.* Channels are split and images are shown in anti-NF/phalloidin **C** and anti-ChAT/phalloidin **C’**, and in the overlay **C”**. (**D–D”**) Whole mount preparations, showing ChAT-negative sensory fibers entering the spindle and forming coils (*arrow* in **D**) within the central region. ChAT-positive motor fibers (*arrows* in **D’**) also enter the spindle. Staining was with anti-NF and anti-ChAT along with GLUT1. Images are shown in NF/anti-GLUT1 **D** and anti-ChAT/anti-GLUT1 **D’** staining, and in the overlay **D”**. (**E**) Bar chart showing the mean distance that ChAT-negative sensory fibers extend across intrafusal fibers. *Scale bars* = 50 µm in **B**; 25 µm in **C** to **C”**, **D** to **D”**; 15 µm in **A** to **A”**. Error bars in bar chart represent standard deviation.

A bundle of axons entered the spindle at the central region (Figs. 2A–A”). In some cases, multiple nerve entry sites were present (see arrows in Fig. 1A). Most axons entering the spindle expressed ChAT (see Figs. 2A–A”). At least one ChAT-negative axon was present at the nerve entry site (see arrows in Figs. 2A–A”). After entry, ChAT-positive motor axons extended toward the muscle spindle's polar region (asterisk in Fig. 2B), whereas ChAT-negative sensory axons coiled around the intrafusal fibers in the central region (see the boxed yellow insets in Fig. 2B, Figs. 2C–C”, 2D–D”). These coils varied between 85 and 638 µm in length (mean value = 210.5 ± 97.7 µm), as measured in the axis of the coils (see the bar chart in Fig. 2E).

**Figure 3.**
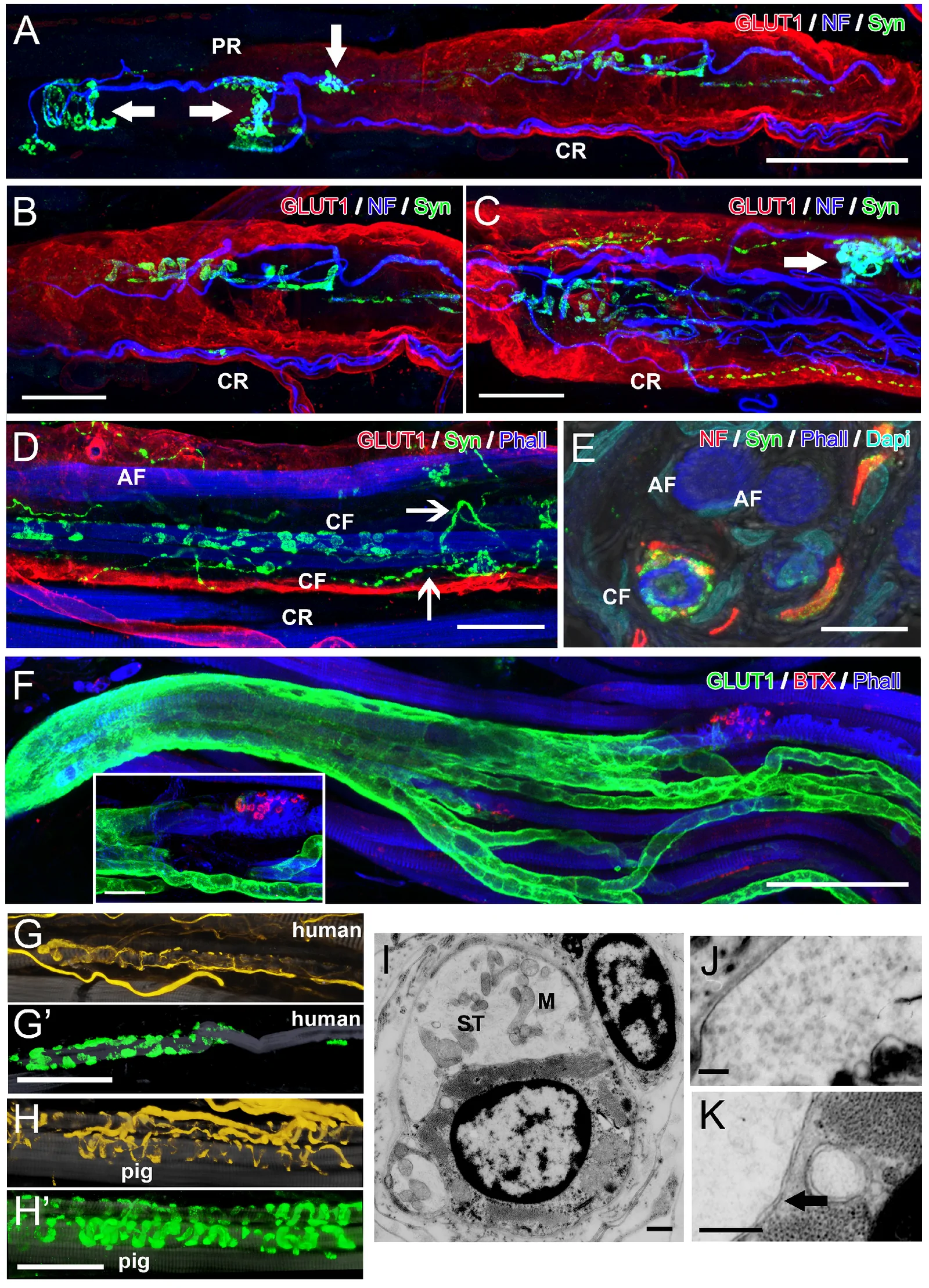
Sensory and motor endings in human EOM spindles. (**A–E**) Confocal microscopy images of muscle spindles showing that sensory and motor nerve terminals express synaptophysin (Syn). **A** to **D** Muscle spindle whole mount preparations showing Syn-positive sensory terminals arranged in a series in the central region (CR). Syn-positive motor terminals are in both the polar region (PR; *arrows* in **A**) and the paracentral region (*arrow* in **C**). Additionally, nerve terminals that are Syn-positive, but not associated with intrafusal fibers (*arrows* in **D**) are present. Labeling was done with anti-GLUT1 (*red*), anti-Syn (*green*), along with anti-NF (*blue*) in **A** to **C** or phalloidin (Phall, *blue*) in **D**. The mixture of *blue* neurofilament and *green* Syn produces a *blue-green* or *cyan* color*.* (**E**) Confocal image of a muscle spindle cross-sectioned in the central region, showing a nuclear chain fiber (CF) with a Syn-positive sensory ending and two anomalous fibers (AFs) without associated Syn-labeled axons. Labeling with anti-NF (*red*), anti-Syn (*green*), and Phall (*blue*), and Dapi (*light blue*). The mixture of *red* neurofilament and *green* Syn produces a *yellowish* color*.* (**F**) Muscle spindle whole mount preparation showing a motor terminal visualized by α-bungarotoxin (BTX) in the muscle spindle's PR. The *boxed inset* shows the motor terminal at higher magnification. Labeling with anti-GLUT1 (*green*), α-bungarotoxin (BTX, *red*), and Phall (*blue*). (**G, G’, H, H’***)* The 3D reconstruction of the sensory axons (labeled with anti-neurofilament: *yellow*) and sensory nerve terminals (labeled with Syn: *green*) in human EOM spindles (**G, G’**) and in pig EOM spindles (**H, H’**). The spatial arrangement shows species-specific differences, with a less elaborate configuration of axons (*yellow*) in humans **G** compared with pigs **H**, and sensory nerve terminals (*green*) exhibiting plaque-like configuration in humans **G’** and an annulo-spiral configuration in pigs **H’**. (**I–K**) Electron micrographs showing a sensory terminal (ST) on a nuclear chain fiber **I**, which contains clear vesicles **J**, and lacks a basal lamina in the synaptic cleft (*arrow* in **K**). Mitochondria (M). *Scale bars* = 100 µm in **A**; 50 µm in **B**, **C**, **D**, **F**, **G**, **G’**, **H**, and **H’**; 25 µm in boxed inset in **F**; 15 µm in **E**; 1 µm in **I**, **J**; 0.5 µm in **K**.

To visualize nerve terminals, we used an antibody against synaptophysin, which labels sensory and motor nerve terminals.^25^ Synaptophysin-positive sensory nerve endings were present in the spindle's central region, where they were arranged in a series (Figs. 3A–C). Synaptophysin-positive sensory contacts were present on all nuclear chain fibers (Figs. 3D, 3E), whereas they were, with a few exceptions, absent on anomalous fibers. Synaptophysin-positive nerve terminals, resembling en-plaque motor terminals, were present in the muscle spindle’s polar region and adjacent to the central region (see the arrows in Figs. 3A, 3C). The motor nature of these terminals was confirmed through labeling with α-bungarotoxin (Fig. 3F), which is a snake venom that binds to acetyl choline receptors in the postsynaptic membrane of motor terminals.^30^ We also observed synaptophysin-positive nerve endings that were not associated with intrafusal muscle fibers (see the arrows in Fig. 3D). These beaded nerve terminals may represent free nerve endings or, alternatively, nerve terminals accompanying blood vessels.

In muscle spindles labeled with antibody against neurofilament and synaptophysin, the spatial arrangement of sensory axons and sensory nerve terminals was analyzed using 3D reconstruction.^32^ Data were then compared with those from pig EOM spindles, given that the sensory innervation in these spindles exhibits typical muscle spindle morphology.^21^ The presence of species-specific differences was observed. Specifically, sensory nerve fibers coiling around intrafusal fibers in human EOM spindles (Fig. 3G) had a less complex arrangement compared with pig EOM spindles (Fig. 3H). Moreover, sensory contacts in human EOM spindles appeared as multiple plaques (Fig. 3G’), whereas their counterparts in pig EOM spindles exhibited the classical annulospiral form (Fig. 3H’).

TEM showed the presence of nerve terminals contacting nuclear chain fibers in the central region of human EOM spindles (Fig. 3I). These nerve terminals contained mitochondria and clear vesicles (see Figs. 3I, 3J). Furthermore, the synaptic cleft between the nerve terminal and the muscle fiber lacked a basal lamina (arrow in Fig. 3K), or other evidence of post-synaptic densities or presynaptic specializations, thereby confirming the sensory characterization of these nerve terminals.^11^

### Sensory Endings in Human EOM Spindles Express Functionally Important Presynaptic Proteins

Sensory endings in skeletal muscle spindles express key proteins at the presynaptic site. These include VGLUT1,^11^^,^^20^^,^^33^ synaptobrevin,^11^ syntaxin,^27^ and complexin.^21^ VGLUT1 facilitates the uptake of the neurotransmitter glutamate into the synaptic vesicles,^20^ whereas synaptobrevin,^11^ syntaxin,^27^ and complexin^28^ are involved in neurotransmitter release. We tested whether these proteins are also present in human EOM spindles.

**Figure 4.**
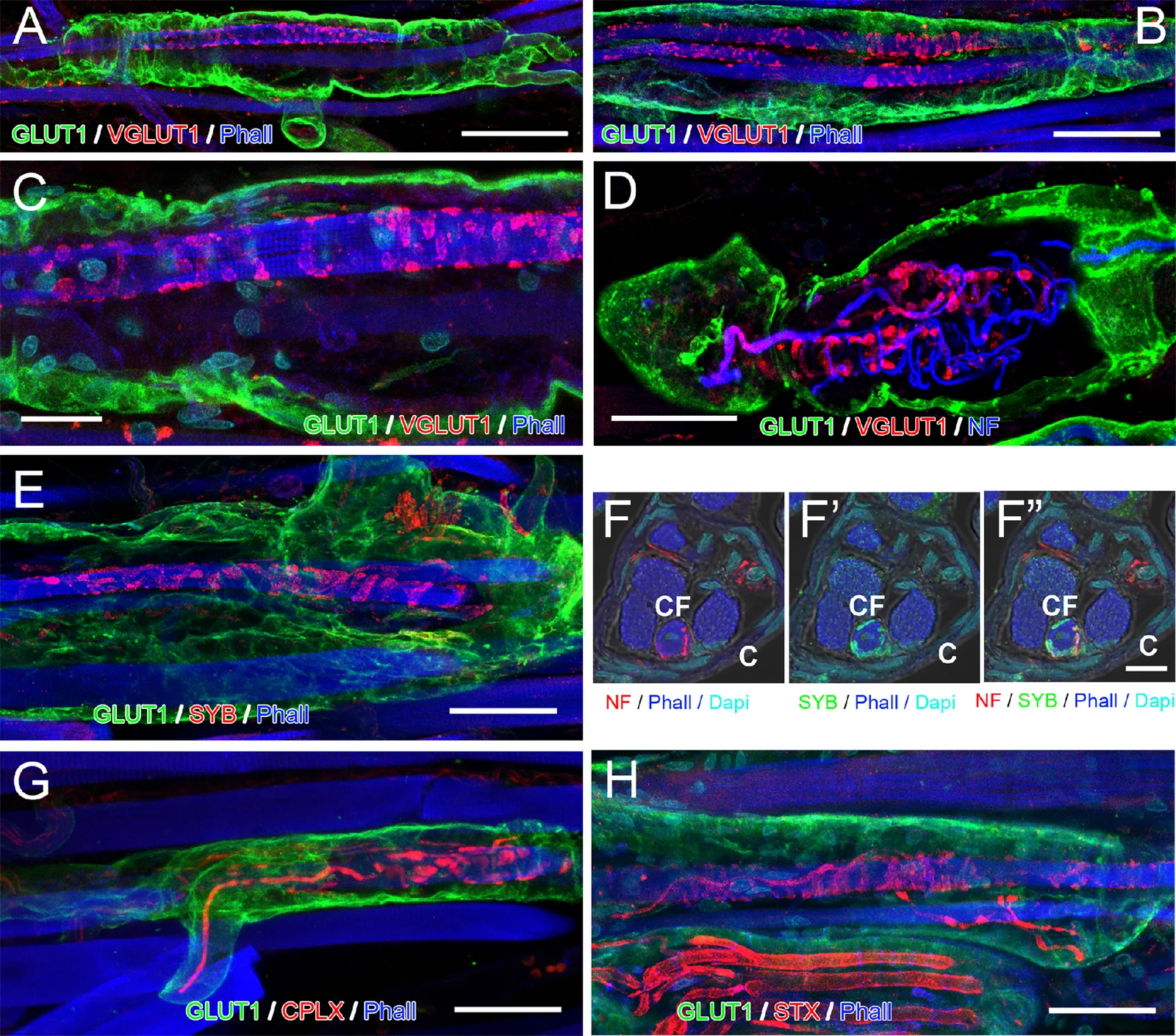
Presynaptic molecules in the sensory nerve terminals of human EOM spindles. (**A–D**) Confocal images of muscle spindle whole mount preparations of the entire muscle spindle (**A, B**) and the central region (**C, D**), showing that sensory endings express VGLUT1. Labeling with anti-GLUT1 (*green*), anti-VGLUT1 (*red*), along with phalloidin (Phall, *blue* in **A–C**) or anti-NF (*blue* in **D**). (**E, F–F”**) Muscle spindle whole mount preparations **E** and cross-sectioned muscle spindles **F** to **F”**, showing synaptobrevin expression in the spindle's sensory endings. In **F** to **F”** synaptobrevin signals are only present on the nuclear chain fiber (CF), but absent in the rest of the intrafusal fibers, which were identified as anomalous fibers. Labeling with anti-GLUT1 (*green* in **E**), anti-synaptobrevin (SYB, *red* in **E***, green* in **F–F”**), along with Phall (*blue* in **E**) or anti-NF (red in **F–F”**). C: spindle capsule. (**G, H**) Confocal images of muscle spindle whole mount preparations, showing complexin **G** and syntaxin **H** expression in the sensory endings. Labeling with anti-GLUT1 (*green*), Phall (*blue*), and anti-complexin (CPLX, *red* in **G**) or anti-syntaxin (STX, *red* in **H**), *Scale bars* = 100 µm in **A**, **B**; 50 µm in **D**, **E**, **G**, and **H**; 25 µm in **C**, and 15 µm in **F** to **F”**

Immunolabeling demonstrated that all four proteins were present in sensory endings of human EOM spindles, which were identified as sensory based on their morphology and association with chain fibers. Figure 4 shows VGLUT1 (Figs. 4A–D), synaptobrevin (Figs. 4E, 4F–F”), complexin (Fig. 4G), and syntaxin immunoreactivity (Fig. 4H) in sensory nerve terminals.

### Proprioceptive Nerve Fibers Do Not Run Within the EOM Motor Nerves

**Figure 5.**
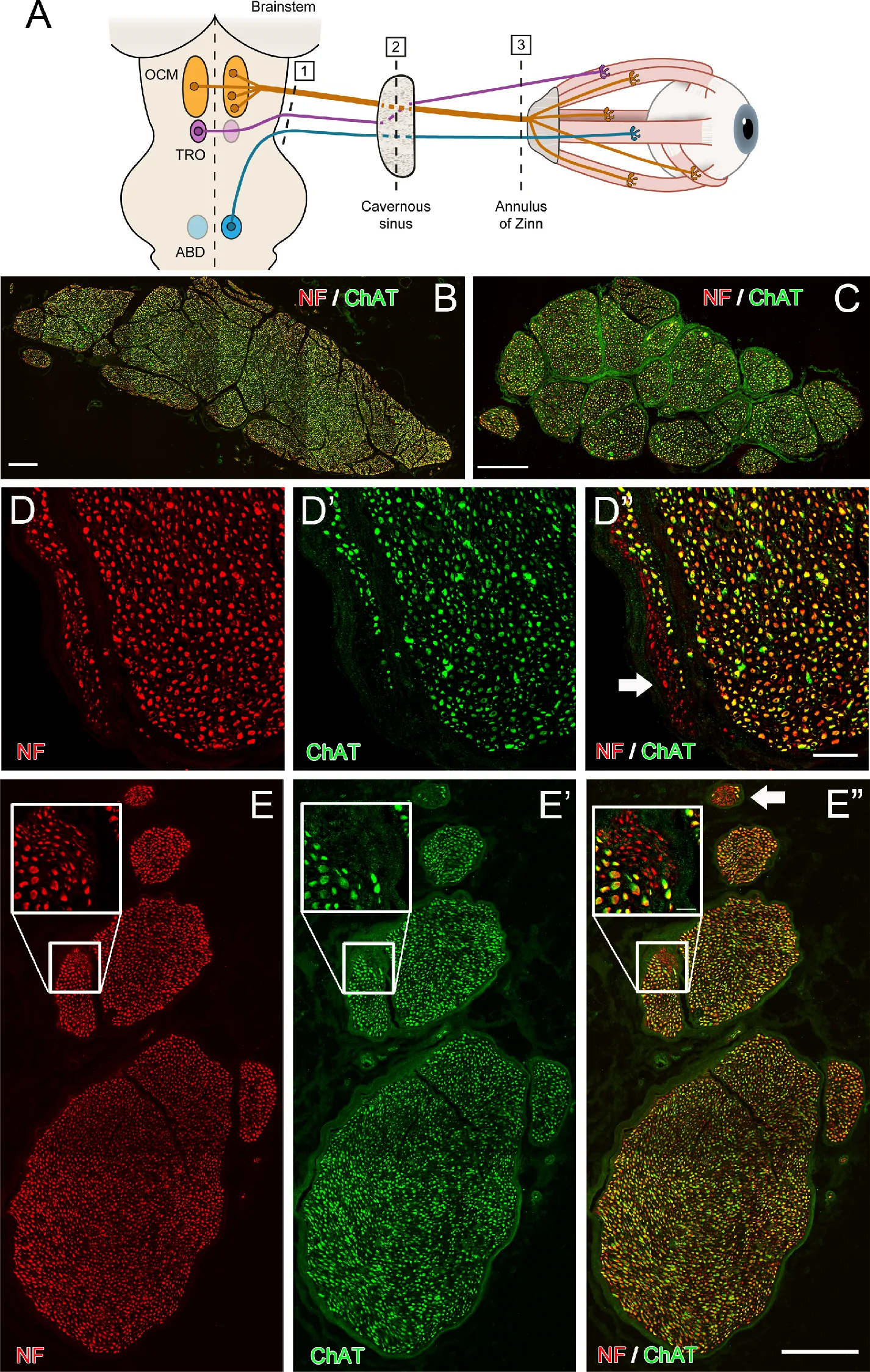
Fiber composition of the EOM motor nerves along its intracranial territory. (**A**) Schematic diagram showing the three sampling sites of the EOM motor nerves. Position 1 is at the exit site from the brain stem (1), position 2 at the mid-level of the cavernous sinus (2), and position 3 at the point of entry into the orbit (3). Oculomotor, trochlear, and abducens nucleus (OCM, TRO, and ABD). (**B**, **C**, **D–D”**, **E–E”**) Confocal images of cross-sectioned oculomotor (**B**, **D–D”**) and abducens nerve (**C**, **E–E”**) at position 1 (**B**, **C**) and position 3 (**D–D”**, **E–E”**) labeled with anti-NF (*red*) and anti-ChAT (*green*). At position 1, all nerve fibers in the oculomotor **B** and abducens nerve **C** are ChAT-positive. Similar findings for position 2 are shown in Supplementary Figure S2. At position 3, both nerves exhibit a mixed fibers composition, with clusters of ChAT-negative fibers (*arrows* in **D”**, *boxed insets* in **E”**). **D** to **D”** shows a detail of the oculomotor nerve, whereas **E** to **E”** shows the entire abducens nerve. *Scale bars* = 200 µm in **B**, **C**, **E**, to **E”**; 50 µm in **D** to **D”**; 15 µm in the *boxed inset* in **E** to **E”**

It has been shown previously that sensory (proprioceptive) fibers originating from human EOM spindles join the EOM motor nerves at the entry site into the muscle.^34^ However, the subsequent trajectory of proprioceptive fibers is uncertain. We tested the hypothesis that these fibers may travel toward the brainstem, integrated in the EOM motor nerves. To this end, samples of the oculomotor, trochlear, and abducens nerve were subjected to ChAT immunolabeling at three positions: at the exit site from the brain stem (position 1; Fig. 5A), at the midlevel of the cavernous sinus (position 2; see Fig. 5A), and at the site where they entered the orbit (position 3; see Fig. 5A).

At position 1 (brain stem exit), all axons of the oculomotor, trochlear, and abducens nerve exhibited ChAT-immunoreactivity (Figs. 5B, 5C). This was also the case at position 2 (cavernous sinus) (Supplementary Fig. S2). At position 3, ChAT-negative axons were present in all three nerves as they entered the orbit. These axons formed either small clusters near the edges of the nerve (Figs. 5D–D”, 5E–E”), or were sometimes in small, separate fascicles near the main nerve (arrow in Fig. 5E”). Most ChAT-negative axons were of medium size (enlargement of the boxed inset in Figs. 5E–E”). To exclude that ChAT-negative axons at position 3 are actually autonomic axons, we performed additional staining with an antibody against TH, which labels postganglionic sympathetic axons.^24^ Labeling with anti-TH revealed that ChAT-negative axons at position 3 lacked TH immunoreactivity (Supplementary Fig. S3). TH-positive axons were only present in thin nerves adjacent to the EOM motor nerves (see Supplementary Fig. S3). Collectively, this suggests that ChAT-negative/TH-negative axons represent sensory (proprioceptive) axons.

## Discussion

**Figure 6.**
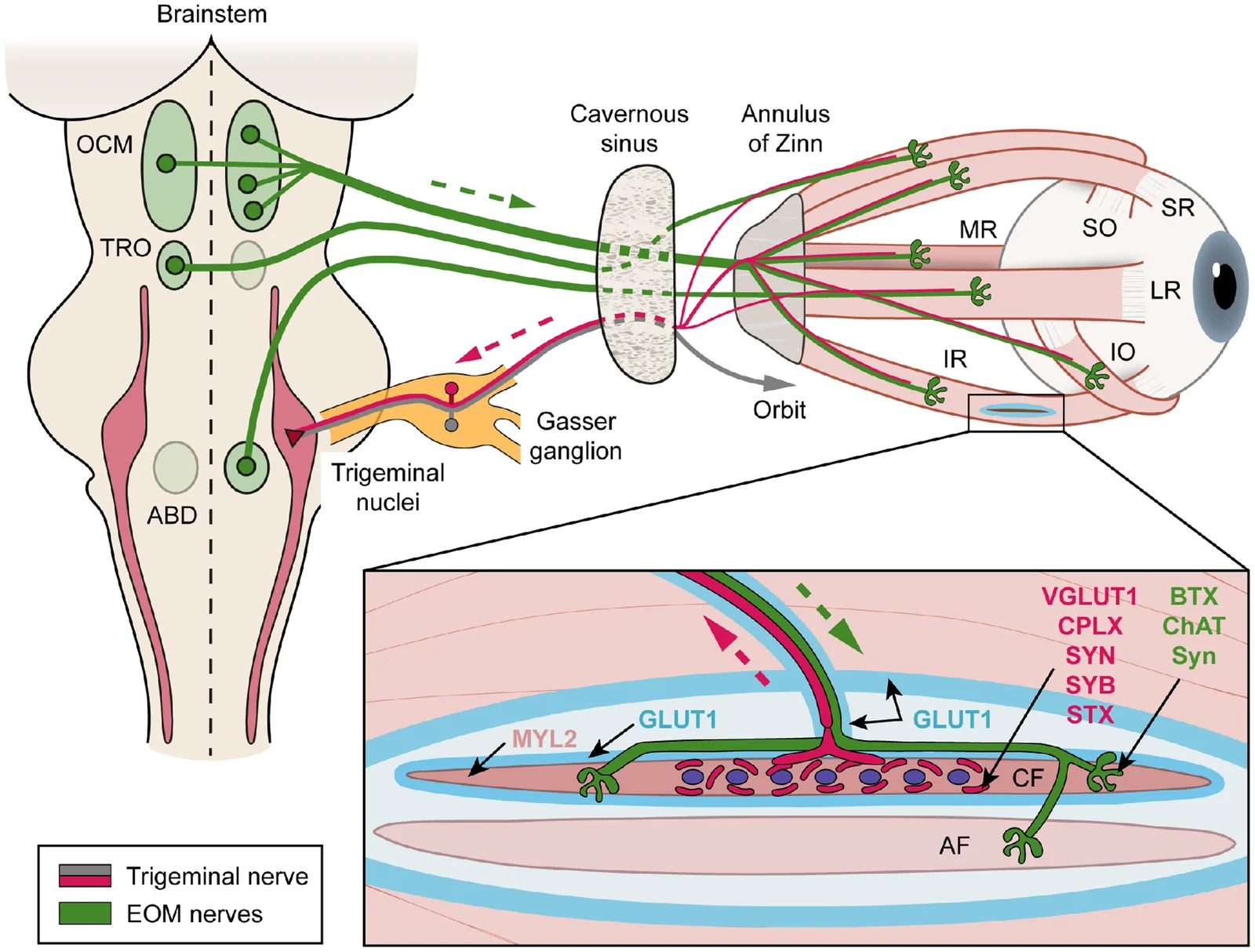
Schematic drawing summarizing the key findings of the present study. Within the EOMs, proprioceptive nerve fibers (*red*) are interwoven with the EOM motor nerves (*green*). Upon exiting the orbit, proprioceptive fibers join the trigeminal nerve (*grey*) to terminate in the trigeminal nucleus. The *large*, *boxed inset* illustrates the molecular characteristics of human EOM muscle spindles, with the outer and inner capsule expressing GLUT1 (*blue*). Nuclear chain fibers (CFs) express MYL2, while anomalous fibers (AFs) do not. Sensory nerve terminals (*red*) express VGLUT1, complexin (CPLX), synaptophysin (SYN), synaptobrevin (SYB), and syntaxin (STX). Motor terminals (*green*) express ChAT, synaptophysin, and α-bungarotoxin (BTX). Superior, inferior, medial, and lateral rectus muscle (SR, IR, MR, and LR). Superior and inferior oblique muscle (SO and IO). Oculomotor, trochlear, and abducens nucleus (OCM, TRO, and ABD).

Human EOMs spindles exhibit unique features, leading to an ongoing debate about their function.^2^^,^^3^^,^^15^^,^^16^ We confirmed that human EOM spindles lack nuclear bag fibers, but do contain nuclear chain fibers along with other fibers, so-called anomalous fibers.^17^ Furthermore, a significant number of fibers do not extend the full length of the spindle.^3^^,^^15^ Unlike two previous studies,^3^^,^^15^ which reported a mean number of eight intrafusal fibers per spindle, a lower number of intrafusal fibers (2.5 ± 1.2) was counted in the present study. This discrepancy can be attributed to a greater sample size, involving 54 muscle spindles in the present study versus 27 in Ruskell's study^17^ and 10 in Blumer's et al. study.^15^ It is specifically due to the relatively high number of muscle spindles that contain only one or two intrafusal fibers, that might have been missed in studies with smaller sample sizes. Through immunofluorescence technology, we have uncovered the molecular signature of human EOM spindles, unveiling the presence of distinct tissue molecules (GLUT1 and MYL2) as well as distinct neuro molecules (VGLUT1, synaptophysin, synaptobrevin, syntaxin, and complexin). We also show that EOM motor nerves do not serve as a pathway for sensory fibers to reach the brain, suggesting the trigeminal nerve is the only plausible route. That the trigeminal nerve is the preferred conduit for proprioceptive fibers to reach the brainstem is consistent with findings in other animal species, using anatomic^35^ and physiological techniques.^36^ A synopsis of the present findings is provided in Figure 6.

Recent finding have shown that the outer capsule of skeletal muscle spindles in mice^19^ and EOM spindles in pigs^21^ express GLUT1. Moreover, the intrafusal fibers in mice skeletal muscle spindles express MYL2.^19^ In line with these studies, GLUT1 was present in the outer capsule of human EOM spindles. GLUT1 was also present in the inner capsule cells that encircled intrafusal muscle fibers. This observation was also made in pig EOM spindles,^21^ but not in skeletal muscle spindles, indicating differences between EOM spindles and skeletal muscle spindles. In skeletal muscle spindles, MYL2 is expressed in nuclear chain fibers and nuclear bag fibers.^19^ In human EOM spindles, MYL2 immunoreactivity was present in nuclear chain fibers, but not in anomalous fibers, indicating molecular differences between these fiber types, which is in line with a previous study.^37^ In conclusion, tissue proteins that are constituents of skeletal muscle spindles and pig EOM spindles, are also present in human EOM spindles, with a few variations in their form of expression.

Consistent with previous studies,^15^^,^^17^ we showed that human EOM spindles receive sensory innervation. We found motor terminals within the EOM spindles of aged persons, which were previously only reported in EOM spindles of a child.^15^ This indicates that sensory and motor innervation in human EOM spindles persists until advanced age, thereby contributing to our understanding of age-related changes in sensory and motor function. It is also in line with previous findings^15^^,^^17^ that sensory innervation is restricted to the nuclear chain fibers, whereas sensory input to anomalous fibers is negligible, suggesting they do not directly supply proprioceptive information. Sensory nerve terminals were arranged as multiple plaques, an organization that differs from the annulospiral configuration in muscle spindles, with canonical morphology. The atypical arrangement of sensory nerve terminals was seen in the EOMs of the aged persons. It was not possible to visualize the 3D arrangement of sensory nerve terminals in the 2-year-old sample, as the spindles were fragmented by sectioning, preventing an overall view of the spindle. It remains to be determined whether the specific configuration of sensory nerve terminals reflects age-related changes, as such changes were observed in skeletal muscle spindles of aged mice,^38^ or if it is a consistent characteristic of human EOM spindles.

The presence of clear vesicles, confirmed by synaptophysin immunolabeling, was a prominent feature of sensory nerve terminals in human EOM spindles. Synaptophysin, a vesicles membrane protein, is supposed to be implicated in the storage and release of neurotransmitters.^25^ Other functionally important molecules at the presynaptic side of sensory nerve endings included VGLUT1, which is necessary for glutamate uptake,^20^ as well as synaptobrevin, syntaxin, and complexin, which are key proteins involved in neurotransmitter release.^26^^–^^28^ Thus, our molecular analyses demonstrate that human EOM spindles possess the molecular machinery for the storage and the release of glutamate. Such a glutamatergic system is also present in skeletal muscle spindles,^11^^,^^20^^,^^33^^,^^39^ and its recent detection in pig EOM spindles^21^ underscores the relevance of this system for muscle spindle function. Glutamate, released from sensory nerve terminals in skeletal muscle, binds to glutamate auto-receptors in the membrane of the same sensory nerve terminal.^11^ This autogenic feedback enhances the sensitivity of the muscle spindle during stretch, indicating that glutamate is vital for spindle function.^33^^,^^39^

The fact that human EOM spindles have an innervation pattern and molecular characteristics consistent with those of skeletal muscle spindles is a strong argument for their functionality and capacity to register length changes in the muscles. However, the absence of nuclear bag fibers suggests that, unlike skeletal muscle spindles, which sense the muscle length changes in terms of both velocity and degree through bag fibers and chain fibers, respectively,^40^ the primary function of human EOM spindles is to sense the degree, rather than the velocity of muscle change. This is consistent with the findings of Goldberg,^8^ who found a tonic eye position signal of proprioceptive origin in the somatosensory cortex of monkeys. Further support for the idea that human EOM spindles contribute to oculomotor control came from a recent study.^5^ Specifically, following manual dislocation of one eye, there was increased neuronal activity in the oculomotor nucleus contralateral to the eye where the muscle was stretched.^5^ This suggests that proprioceptive information produced by muscle stretch and likely sensed by the muscle spindles described here does lead to activity changes throughout the extraocular motor system. Furthermore, it is possible that, analogous to skeletal muscle spindles, glutamate release contributes to an increased sensitivity of human EOM spindles, which might be relevant for the fine-tuning of eye movements to optimize visual acuity. The function of anomalous fibers in human EOM spindles remains to be elucidated. Furthermore, it is not clear why some intrafusal fibers do not extend all the way to the end of the spindle.

It should be noted that it is unlikely that proprioceptive signals derive from palisade endings, which are unique nerve specializations in mammalian including human EOMs.^41^^–^^43^ Due to their distinct location at the myotendinous junction, palisade endings have been considered to be sensory receptors for many years,^44^^–^^47^ providing eye position signals. However, the sensory role of palisade endings has been put into question as recent studies in cats^48^ and monkeys^49^^,^^50^ have shown that these structures are cholinergic and originate from the EOM motor nuclei.

The value of EOM proprioception in oculomotor control is still a matter of debate.^2^ One of the reasons is that the endowment of muscle spindles within the EOMs varies across mammalian species.^51^ Muscle spindles are a constant feature in the EOMs of even-toed ungulates, where they occur in high numbers.^51^^,^^52^ In case of primate EOMs, muscle spindles are present in both humans and in monkeys, with a significantly lower number in monkeys compared with humans.^51^^,^^53^ In the rest of the species studied to date, muscle spindles are absent in the EOMs.^51^ The enigma surrounding due to the presence or absence of muscle spindles in the EOMs of various species remains unresolved. It is possible that the judgment of gaze direction might be an integration of the internal representation of the corollary discharge with proprioceptive signals from multiple sources, including the vestibular system,^54^ general proprioceptors in the neck muscles,^55^ and the orbit itself, including the eye muscles with the main corrective assistance of vision.^56^ Finally, the integration of the spinal central pattern generator for locomotion with gaze control is of paramount importance during walking.^57^^,^^58^

In summary, the present study has identified the molecular signature of human EOM spindles, thereby supporting the hypothesis that they function as stretch receptors, providing the tonic eye position proprioceptive signal that has been found electrophysiologically in the somatosensory cortex of primates.^8^ These novel insights will definitively advance our understanding of the interplay between eye movements and sensory feedback, and they support the hypothesis that proprioception from EOMs is important for eye position information.

## Supplementary Material

Supplement 1

Supplement 2
