# Supplementary material for: Structural and Molecular Features of Human Extraocular Muscle Spindles Suggest a Unique Role in Proprioception
Source: Invest Ophthalmol Vis Sci. 2026 Sep 15;67(11):27. doi: 10.1167/iovs.67.11.27 (PMC13589405; doi:10.1167/iovs.67.11.27)
Supplement: Supplementary file 1 [file iovs-67-11-27_s001.docx]

**Supplementary Figure 1**

A series of cross sections through the muscle spindle, presented in Figures 1G and 1H, is shown in different staining combinations. The spindle has initially four intrafusal fibers (A1 – A4), with a fiber stopping at A5 (arrow). Figure A7 shows the muscle spindle’s polar region. Muscle fibers are not labeled with phalloidin and are instead visualized by bright field technique. Intrafusal fibers, of which some have already departed the spindle, are highlighted with an "IF" label. The distance between individual sections is 50 µm from A1 to A6 and 400 µm from A6 to A7. Scale bars: 15 µm

**Supplementary Figure 2**

Fiber composition of the oculomotor and the abducens nerve at position 2 (see Fig. 5A) of the sampling site.

Confocal images of cross-sectioned oculomotor (**A – A”**) and abducens nerve (**B – B”**) at position 2 labeled with anti-NF (red) and anti-ChAT (green). All nerve fibers in the oculomotor (**B**) and abducens nerve (**C**) are ChAT-positive. Scale bars: 200 µm in **A – A”**, **B – B”**

**Supplementary Figure 3**

Section through the abducens nerve at position 3 (see Fig. 5A). **A***,* **B – B***”* Labeling was with in anti-NF (red)/anti-TH (green). TH-positive postganglionic sympathetic axons are virtually absent in the sample containing the abducens nerve, except a few TH-positive fibers in a single fascicle near the main nerve (arrow in **A**). This fascicle is shown at higher magnification in **B – B”**. The nerve fibers encircled with white lines in *A* correspond to clusters of ChAT-negative fibers, which are shown in Figure 5E - 5E” in a neighboring section. Scale bars: 200 µm in **A** and 15 µm in **B – B”**
