# Supplementary material for: Structural and Molecular Features of Human Extraocular Muscle Spindles Suggest a Unique Role in Proprioception
Source: Invest Ophthalmol Vis Sci. 2026 Sep 15;67(11):27. doi: 10.1167/iovs.67.11.27 (PMC13589405; doi:10.1167/iovs.67.11.27)
Supplement: Supplementary file 2 [file iovs-67-11-27_s002.docx]

**Supplemental Table 1.** Comprehensive list of human EOM spindles identified and included in this study. Each spindle was assigned a ‘spindle number’ for reference. Muscles included were the inferior rectus (*IR*), medial rectus (*MR*), and inferior oblique (*IO*). Staining types varied depending on how the tissue was processed, either as cross sections (*cs*) or whole mount sections (*wm*). Mean and standard deviation (*sd*) are listed at the bottom of the table.

| **Spindle Number** | **Muscle** | **Staining type** | **Total fiber number**  **(TF)** | **Chain fibers (CF)** | **Bag fibers**  **(BF)** | **Anomalous fibers**  **(AF)** | **Spindle diameter (µm)** | **Capsule length**  **(µm)** | **Number**  **fibers w/**  **sensory terminals** | **Length of sensory innervation (µm)** | **Chain fiber diameter (µm)** | **Anomalous fiber diameter**  **(µm)** |
| --- | --- | --- | --- | --- | --- | --- | --- | --- | --- | --- | --- | --- |
| 1 | IO | cs | 4 | 2 | 0 | 2 | 79.7 | 1200 | 2 |  | 6.1  9.31 | 9.3  14.2 |
| 2 | IO | cs | 3 | 2 | 0 | 1 | 59.3 | 900 | 2 |  | 11.5  13.5 | 14.3 |
| 3 | IR | cs | 2 | 1 | 0 | 1 | 48.9 | 1200 | 1 |  | 13.7 |  |
| 4 | IR | cs | 2 | 1 | 0 | 1 | 88.9 | 600 | 1 |  | 14.2 | 15.5 |
| 5 | MR | wm | 2 | 1 | 0 | 1 |  | 1000 | 1 | 150 |  |  |
| 6 | MR | wm | 2 | 1 | 0 | 1 |  | 690 | 2 |  |  |  |
| 7 | IR | cs | 3 | 1 | 0 | 2 | 79.1 | 630 | 1 | 160 | 11.3 | 9.9  15.4 |
| 8 | IR | cs | 3 | 2 | 0 | 1 | 47.8 |  | 2 |  | 13.0  10.6 | 13.6 |
| 9 | IR | cs | 1 | 1 | 0 | 0 | 71.4 | 550 | 1 |  | 12.9 |  |
| 10 | IR | cs | 3 | 1 | 0 | 2 | 89.0 | 500 | 1 |  | 14.1 | 22.3  25.4 |
| 11 | IR | cs | 2 | 2 | 0 | 0 | 67.8 | 360 | 2 |  | 7.0  14.6 |  |
| 12 | IR | cs | 4 | 4 | 0 | 0 | 94.6 | 600 | 4 | 240 | 9.6  8.8  10.0  9.3 |  |
| 13 | IR | cs | 5 | 3 | 0 | 2 | 69.9 |  | 3 |  | 8.4  6.8  9.0 | 12.4  20.2 |
| 14 | IR | cs | 4 | 1 | 0 | 3 | 83.8 |  | 1 |  | 13.0 | 12.5  11.9  14.5 |
| 15 | IR | cs | 1 | 1 | 0 | 0 | 40.4 |  | 1 |  | 10.2 |  |
| 16 | IR | cs | 5 | 1 | 0 | 4 | 71.5 | 700 | 1 | 180 | 12.2 | 14.5  13.8  16.9  8.1 |
| 17 | IR | cs | 2 | 1 | 0 | 1 | 59.6 | 800 | 1 |  | 9.2 | 16.0 |
| 18 | IO | wm | 1 | 1 | 0 | 0 |  |  | 1 | 250 |  |  |
| 19 | IR | cs | 2 | 1 | 0 | 1 | 54.8 | 280 | 1 |  | 7.9 | 13.1 |
| 20 | IO | cs | 2 | 1 | 0 | 1 | 48.4 | 600 | 1 |  | 10.0 | 9.7 |
| 21 | IO | cs | 4 | 1 | 0 | 3 | 70.5 | 810 | 1 |  | 7.3 | 24.5  13.8  13.5 |
| 22 | MR | wm | 5 | 2 | 0 | 3 | 127.4 | 563.2 | 2 | 145.3 |  |  |
| 23 | MR | wm | 2 | 1 | 0 | 1 | 91.1 | 828 | 1 | 192.7 |  |  |
| 24 | MR | wm | 4 | 2 | 0 | 2 | 125.3 | 1080.1 | 2 | 230.4 |  |  |
| 25 | MR | wm | 5 | 3 | 0 | 2 | 109.5 | 845.5 | 3 | 161.0  285.1  245.1 |  |  |
| 26 | MR | wm | 2 | 1 | 0 | 1 | 58.7 | 708.8 | 1 | 459.3 |  |  |
| 27 | MR | wm | 1 | 1 | 0 | 0 |  |  | 1 |  |  |  |
| 28 | MR | wm | 2 | 1 | 0 | 1 |  |  | 1 |  |  |  |
| 29 | MR | wm | 3 | 2 | 0 | 1 | 84.0 | 967.7 | 2 | 215.2  105.3 |  |  |
| 30 | MR | wm | 3 | 2 | 0 | 1 |  |  | 2 | 193.3  163.6 |  |  |
| 31 | MR | wm |  |  |  |  | 81.9 | 620.7 |  | 146.3 |  |  |
| 32 | MR | wm |  |  |  |  | 66.6 |  |  | 141.2  150.0 |  |  |
| 33 | MR | wm |  |  |  |  | 100.8 | 787.1 |  | 132.2 |  |  |
| 34 | MR | wm | 3 | 2 | 0 | 1 | 102.1 | 1114.7 | 2 | 638.5  250.4 |  |  |
| 35 | MR | wm | 3 | 2 | 0 | 1 | 93.2 | 845.4 | 2 | 248.0  136.0 |  |  |
| 36 | MR | wm | 3 | 1 | 0 | 2 | 61.3 | 522.4 | 1 |  |  |  |
| 37 | MR | wm | 1 | 1 | 0 | 0 | 54.0 |  | 1 |  |  |  |
| 38 | MR | wm | 1 | 1 | 0 | 0 | 81.9 | 766.9 | 1 | 168.4 |  |  |
| 39 | MR | wm | 3 | 3 | 0 | 0 | 69.6 |  | 3 |  |  |  |
| 40 | MR | wm | 2 | 2 | 0 | 0 | 58.4 |  | 2 |  |  |  |
| 41 | MR | wm | 1 | 1 | 0 | 0 | 45.6 | 581.2 | 1 | 290.7 |  |  |
| 42 | MR | wm | 1 | 1 | 0 | 0 | 78.8 | 1046.1 | 1 | 357.1 |  |  |
| 43 | MR | wm | 1 | 1 | 0 | 0 | 58.8 |  | 1 | 195.4 |  |  |
| 44 | IR | wm | 2 | 1 | 0 | 1 | 79.0 | 477.5 | 1 | 231.4 |  |  |
| 45 | IR | wm | 3 | 3 | 0 | 0 | 116.9 | 838.4 | 3 | 289.5  160.7  179.5 |  |  |
| 46 | IR | wm | 2 | 2 | 0 | 0 | 58.9 | 701.3 | 2 | 210.6  143.8 |  |  |
| 47 | IR | wm | 2 | 2 | 0 | 0 | 69.4 | 608.1 | 2 | 149.2  150.4 |  |  |
| 48 | IR | wm | 1 | 1 | 0 | 0 | 40.6 | 627.7 | 1 | 172.9 |  |  |
| 49 | IR | wm | 4 | 2 | 0 | 2 | 97.6 |  | 2 | 221.3 |  |  |
| 50 | IR | wm | 2 | 1 | 0 | 1 | 93.1 |  | 1 | 249.1 |  |  |
| 51 | IR | wm | 3 | 3 | 0 | 0 | 91.6 |  | 3 | 182.5 |  |  |
| 52 | IR | wm |  |  |  |  | 91.6 |  | 2 | 84.9  99.1 |  |  |
| 53 | IR | wm |  |  |  |  | 134.3 |  | 1 | 295.7 |  |  |
| 54 | IR | wm | 2 | 2 | 0 | 0 | 96.2 |  | 2 |  |  |  |
|  | | **Mean** | **2.5** | **1.6** | **0.0** | **1.0** | **78.0** | **741.5** | **1.6** | **210.5** | **10.5** | **14.8** |
|  |  | *sd* | *1.2* | *0.8* | *0.0* | *1.0* | *22.8* | *224.5* | *0.8* | *97.7* | *2.5* | *4.4* |
